# Structure and properties of Co-doped ZnO films prepared by thermal oxidization under a high magnetic field

**DOI:** 10.1186/s11671-015-0834-2

**Published:** 2015-03-07

**Authors:** Guojian Li, Huimin Wang, Qiang Wang, Yue Zhao, Zhen Wang, Jiaojiao Du, Yonghui Ma

**Affiliations:** Key Laboratory of Electromagnetic Processing of Materials (Ministry of Education), Northeastern University, No 3-11, Wenhua road, Heping district Shenyang, 110819 China

**Keywords:** High magnetic field, ZnO film, Oxidation, Thin film

## Abstract

The effect of a high magnetic field applied during oxidation on the structure, optical transmittance, resistivity, and magnetism of cobalt (Co)-doped zinc oxide (ZnO) thin films prepared by oxidizing evaporated Zn/Co bilayer thin films in open air was studied. The relationship between the structure and properties of films oxidized with and without an applied magnetic field was analyzed. The results show that the high magnetic field obviously changed the structure and properties of the Co-doped ZnO films. The Lorentz force of the high magnetic field suppressed the oxidation growth on nanowhiskers. As a result, ZnO nanowires were formed without a magnetic field, whereas polyhedral particles formed under a 6 T magnetic field. This morphology variation from dendrite to polyhedron caused the transmittance below 1,200 nm of the film oxidized under a magnetic field of 6 T to be much lower than that of the film oxidized without a magnetic field. X-ray photoemission spectroscopy indicated that the high magnetic field suppressed Co substitution in the ZnO lattice, increased the concentration of oxygen vacancies, and changed the chemical state of Co. The increased concentration of oxygen vacancies affected the temperature dependence of the resistivity of the film oxidized under a magnetic field of 6 T compared with that of the film oxidized without a magnetic field. The changes of oxygen vacancy concentration and Co state caused by the application of the high magnetic field also increase the ferromagnetism of the film at room temperature. All of these results indicate that a high magnetic field is an effective tool to modify the structure and properties of ZnO thin films.

## Background

Diluted magnetic semiconductors (DMS) show potential for spintronics, magneto-optical, optoelectronic, and magneto-electronic applications because of the coexistence of optical, electrical, and magnetic properties and also their high-predicted Curie temperature [1,2]. Cobalt (Co)-doped zinc oxide (ZnO) is one of the most promising candidates as a DMS because of its wide direct bandgap (3.37 eV), high exciton binding energy (60 meV), low cost, non-toxicity, and stability over a wide temperature range [3-5]. Doping ZnO with Co can vary its local structure, vacancies, and interstitial atoms and is an effective method to modify the electrical, optical, and magnetic properties of ZnO films [6-10]. The main limitations of Co-doped ZnO that need to be overcome to promote its use in practical applications is to increase its Curie temperature above room temperature (RT) and find the origin of its magnetism.

To date, the origin of the magnetism in Co-doped ZnO has been debated, which hinders the further manipulation of its properties. Defect states, secondary phases, and Co clusters may play important roles in determining the magnetic properties of this material [11-13]. Apart from its magnetic properties, the optical transmittance and electrical conductivity of Co-doped ZnO DMS may be useful for various applications. Generally, the structure and properties of transition metal-doped ZnO strongly depend on the growth and preparation conditions [14-16].

In our previous studies [17-19], we found that application of an *in situ* high magnetic field can change phase formation at the nanoscale, improving the soft magnetic properties of Ni-Fe films and inducing pillar growth of Co films without changing the composition or growth conditions. In this study, a new method to prepare Co-doped ZnO films by oxidizing an evaporated Zn/Co bilayer thin film under a high magnetic field is proposed. The surface morphology, crystal structure, chemical states, magnetism, optical transmittance, and electrical resistivity of the resulting materials are studied to explore their structure–property relationships and define the effect of application of a high magnetic field during oxidation.

## Methods

Zn/Co bilayer thin films were evaporated by the molecular beam vapor deposition method described in our previous studies [19-21]. The base pressure was better than 8.0 × 10^−5^ Pa and changed to about 1.2 × 10^−4^ Pa during deposition. The substrate was a polished quartz plate with a thickness of 0.8 mm that was ultrasonically cleaned in acetone and then alcohol for 15 min and finally dried by an argon gun at ultra-high pressure before use. The substrate was maintained at RT during deposition. High-purity Zn (99.99%) and Co (99.995%) particles with a diameter of about 3 mm were used as the source materials.

Zn/Co bilayer thin films were fabricated in three steps. First, a Co film with a thickness of 10 nm was deposited on the substrate as the doping source by evaporating Co for 10 min at 1,390°C. Second, a Zn film with a thickness of 280 nm was deposited on the Co film by evaporating Zn for 20 min at 350°C. Finally, the Zn/Co bilayer thin films were oxidized in open air for 30 min at 500°C with a heating rate of 5°C/min in a heat-treatment furnace under a superconducting magnet (JMTD-12 T-100) with the magnetic flux densities of 0 and 6 T. The direction of the magnetic field was upward and perpendicular to the substrate.

The surface morphology and thickness of the samples were examined by scanning electron microscopy (SEM; SUPRA 35, Carl Zeiss Inc., Oberkochen, Germany) and atomic force microscopy (AFM; Multimode III, Veeco Instruments, Inc., NY USA). The content and chemical state of elements was determined by energy-dispersive X-ray spectroscopy (EDX; Inca, Oxford Instruments, Abingdon, UK) and X-ray photoemission spectroscopy (XPS; ESCALAB 250Xi, Thermo Fisher Scientific, Waltham, MA, USA). The structure of the films was examined by X-ray diffraction (XRD; DMAX 2400, Rigaku, Shibuya-ku, Japan) with a grazing incidence of 2° in *θ*-2*θ* mode with monochromatic Cu Kα1 radiation (*λ* = 0.154056 nm) and transmission electron microscopy (TEM, 2100 F, JEOL Ltd., Akishima-shi, Japan). Optical transmittance was recorded with a UV–vis spectrophotometer (Lambda 750S, PerkinElmer, Waltham, MA, USA). Electrical resistivity was measured by using Keithley equipment (Keithley Instruments, Inc., Cleveland, OH, USA). Magnetic properties were evaluated by a vibrating sample magnetometer (VSM; Lake Shore 7407; Lake Shore Cryotronics, Inc., Westerville, OH, USA).

## Results and discussion

**Figure 1:**
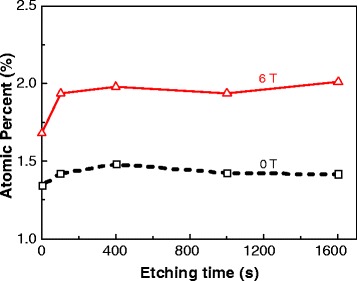
**XPS depth profiles.** Distribution of Co in the thin films oxidized under magnetic fields of 0 and 6 T determined by XPS.

XPS revealed that the Co contents of the Co-doped ZnO thin films oxidized under magnetic fields of 0 and 6 T were 1.48% and 1.97%, respectively, while those measured by EDX were 1.43% and 1.54%, respectively. The difference between the results obtained by XPS and EDX may be caused by the limited accuracy of the equipment. However, these values do confirm that the Co content of the film oxidized under a high magnetic field is slightly higher than that of the film oxidized without a magnetic field. This may be because the high magnetic field promotes the diffusion of Co. Additionally, the depth profiles obtained by XPS indicate that Co is distributed uniformly in both films, as shown in Figure 1. This means that the method used in this study is effective to fabricate Co-doped ZnO films.

**Figure 2:**
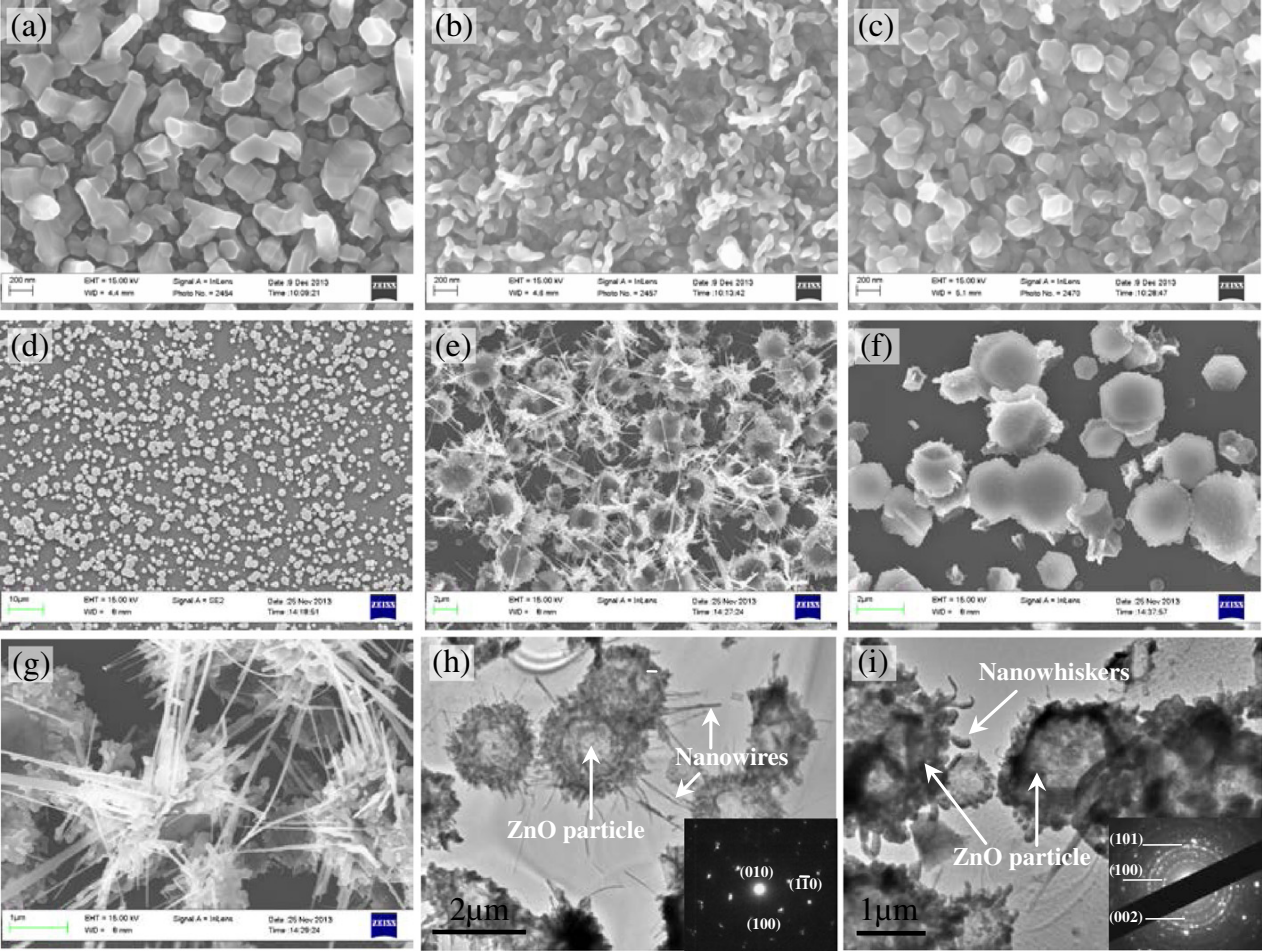
**Morphology of samples.** SEM images of **(a)** as-deposited Zn/Co bilayer film, **(b)** Co-doped ZnO film oxidized without an applied magnetic field, **(c)** Co-doped ZnO film oxidized under an applied magnetic field of 6 T, **(d)** Zn particles deposited at 460°C for 15 min on Si (100), **(e)** ZnO particles formed by oxidation of the particles in **(d)** at 500°C for 40 min without an applied magnetic field, **(f)** ZnO particles formed by oxidation of the particles in **(d)** at 500°C for 40 min under an applied magnetic field of 6 T, **(g)** high-magnification view of **(e)**. TEM images of the ZnO particles oxidized under applied magnetic fields of **(h)** 0 T and **(i)** 6 T. Insets are the corresponding selected area electron diffraction patterns.

**Figure 3:**
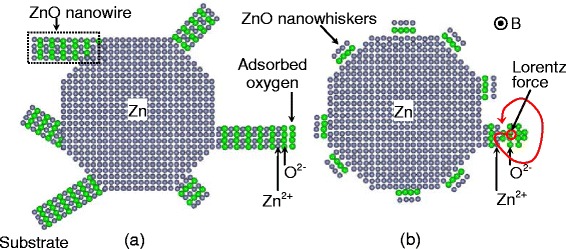
**Schematic of the formation mechanism of ZnO by thermal oxidation under different magnetic field conditions.** Formation of ZnO under applied magnetic fields of **(a)** 0 T and **(b)** 6 T.

The surface morphologies of the as-deposited Zn and Co-doped ZnO films are presented in Figure 2. The surface of the as-deposited Zn film consisted of polyhedral particles. After the Zn was oxidized to ZnO, the morphology of the films changed considerably depending on whether or not a high magnetic field was applied. The film oxidized without an applied magnetic field comprised dendrite-like particles, while that oxidized under a magnetic field of 6 T contained polyhedral particles. To define the effect of the high magnetic field during film oxidation, Zn particles were prepared by evaporating Zn for 15 min at 460°C on Si (100), as shown in Figure 2d. The diameter of these particles was about 1 μm. Then, the particles were oxidized for 40 min at 500°C under magnetic fields of 0 and 6 T. The surface morphologies of the resulting particles are depicted in Figure 2e,f,g. The ZnO particles oxidized without an applied magnetic field are connected by nanowires with a length of about several micrometers and diameter of about 20 nm. In contrast, the ZnO particles oxidized under a magnetic field of 6 T were polyhedral. To further confirm the effect of a high magnetic field on the oxidation of Zn, TEM was used to examine the structure of the ZnO particles, as shown in Figure 2h,i. Although some of the nanowires may have been destroyed by ion milling and polishing, we still can see that the particles oxidized without an applied magnetic field were connected by nanowires, and only polyhedral particles were retained for sample oxidized under a magnetic field of 6 T. These results are consistent with those of SEM. The selected area electron diffraction patterns of the samples indicate that the particles oxidized without an applied magnetic field were mainly *c*-axis oriented, while those oxidized under an applied magnetic field of 6 T were polycrystalline. This is because crystal growth was affected by the Lorentz force of the high magnetic field, as discussed below. Additionally, for the ZnO particles oxidized under a high magnetic field, nanowhiskers appear on the surface of the particles. This means that the high magnetic field suppresses the growth of long nanowires at the apexes of the Zn particles. In the fabrication of ZnO by thermal oxidation of metallic Zn, oxygen in the air is first adsorbed onto the Zn atoms. The neutral Zn atoms then lose electrons and become ions. Finally, the released electrons reduce oxygen molecules to oxygen ions at the metal-oxide interface. The ZnO nanowires are formed by the diffusion of oxygen and Zn ions, as shown in Figure 3a. ZnO nanowires tend to grow at the apexes of Zn particles [22]. Application of a 6 T magnetic field causes the diffusion of oxygen ions to be affected by a Lorentz force (*q****v*** × ***B***) [23] that is perpendicular to both the velocity of ions and magnetic field, as illustrated in Figure 3b. This force suppresses the growth of ZnO at the apexes of Zn particles. As a result, ZnO nanowhiskers formed on the surface of the Zn particles instead the ZnO nanowires. Therefore, the presence of a high magnetic field during oxidation leads to the formation of polyhedral particles.

**Figure 4:**
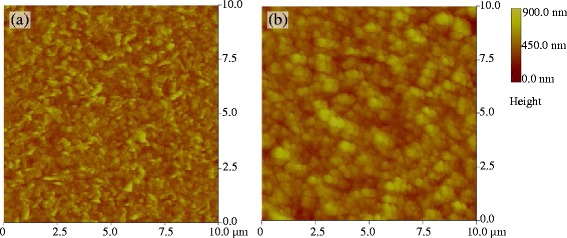
**AFM images of films oxidized with and without an applied magnetic field.** Co-doped ZnO films oxidized under applied magnetic fields of **(a)** 0 T and **(b)** 6 T.

AFM was also used to observe the morphology of the Co-doped ZnO films (Figure 4). In both films, the particles are compactly arranged. Additionally, the morphologies of the films are consistent with the results of SEM. The surface of the film oxidized without an applied magnetic field consisted of dendrite-like aggregates of small particles. The surface of the film oxidized under a magnetic field of 6 T was composed of polyhedral particles that were much larger than those in the film oxidized without an applied magnetic field.

**Figure 5:**
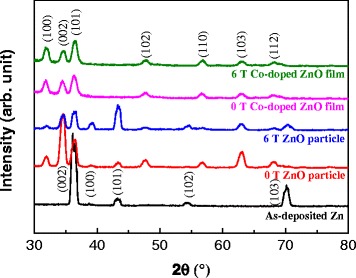
**XRD patterns of the samples oxidized with and without an applied magnetic field.**

XRD was used to investigate the crystal structures of the films (Figure 5). The crystal structure of the as-deposited Zn film was hexagonal with (002) preferred orientation. When the Zn particles were oxidized to ZnO, their peaks changed considerably. The (101), (002), and (100) peaks of ZnO appeared in the diffraction patterns of the particles oxidized under applied magnetic fields of both 0 and 6 T. Several peaks of Zn still remained, but they differed between the oxidized samples. For the ZnO formed without an applied magnetic field, only the (101) peak of Zn obviously remained. Meanwhile, for ZnO oxidized under an applied magnetic field of 6 T, the (100), (101), (102), and (103) peaks of Zn were still visible. This means that the oxidation of Zn was not complete under a 6 T magnetic field, which is because a Lorentz force from the high magnetic field acted on the oxygen ions. As a result, a high magnetic field suppresses the oxidation of Zn. Additionally, the preferred orientations of ZnO particles are also different. ZnO formed without an applied magnetic field has (002) preferred orientation, while those for the sample oxidized under a magnetic field of 6 T are (101) for ZnO and (101) for Zn. In contrast, the Co-doped ZnO films did not contain Zn peaks in their XRD patterns, and the peak positions were consistent with hexagonal wurtzite crystal structure. Furthermore, the preferred orientations for both films were (101). Because the Co content of the film was slightly increased under the high magnetic field, the peak position shifted to higher angle, which decreased the lattice parameter *c*. The Co-doped ZnO films oxidized under magnetic fields of 0 and 6 T exhibited *c* of 0.5195 and 0.5180 nm, respectively. Additionally, the full width at half-maximum (FWHM) of the three strongest peaks for the films oxidized under magnetic fields of 0 and 6 T were 0.73 ± 0.06° and 0.76 ± 0.03°, respectively. The grain sizes in these films were then calculated from their FWHM to be 11.84 ± 0.9 and 11.42 ± 0.7 nm for the films oxidized under magnetic fields of 0 and 6 T, respectively. This suggests that the grain sizes of both films are similar, even though AFM indicated that the surface particle size of the film oxidized under a magnetic field of 6 T was larger than that of the film oxidized without an applied magnetic field.

**Figure 6:**
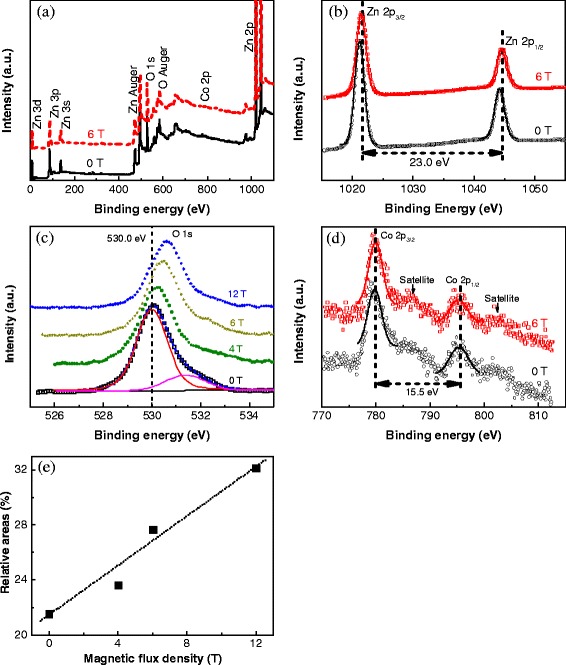
**XPS spectra of Co-doped ZnO films. (a)** XPS survey scan spectra. High-resolution XPS of **(b)** Zn 2p, **(c)** O 1 s, and **(d)** Co 2p state energies. **(e)** Dependence of magnetic flux density on the relative area of oxygen vacancies versus O ions in **(c)**.

XPS was used to explore the chemical states of elements. The XPS survey scan spectra in Figure 6a show that all peaks could be assigned to Zn, O, and Co and no other impurities were observed in the films. The Zn 2p spectra in Figure 6b contain two peaks identified as Zn 2p3/2 and Zn 2p1/2. The difference in binding energy between these peaks is about 23.00 eV for both films and consistent with that of ZnO [24]. The presence of Co in the ZnO films causes the binding energy of Zn 2p to shift to lower binding energy. Furthermore, a higher Co content leads to a larger shift of binding energy. In this study, the shift of the film oxidized without an applied magnetic field is larger than that of the film oxidized under an applied magnetic field of 6 T. This indicates that more Zn atoms were substituted by Co in the film oxidized without an applied magnetic field. The O 1 s spectra of the films (Figure 6c) both contain a peak at about 530.0 eV, although the binding energy of the film oxidized under an applied magnetic field of 6 T appears at higher value, so it contains more oxygen vacancies than that oxidized without an applied magnetic field [10].

The high-resolution spectra of oxygen were deconvoluted to study the relation between oxygen vacancies in the films and magnetic flux densities. The high-resolution spectra of oxygen contained a low-energy peak at 530.0 eV related to the intrinsic binding energy of the O ion in ZnO and a high-energy peak at 531.6 eV originating from oxygen vacancies. The concentration of oxygen vacancies was determined by fitting the high-resolution O 1 s spectra using two Gaussian functions, as shown in Figure 6c. The vacancy concentration is denoted by the relative area of the fitting curve under the high-energy peak versus that of the peak at low binding energy. The shift of binding energy to higher value increases with magnetic flux density. The relationship between the concentration of oxygen vacancies and magnetic flux density according to relative area is plotted in Figure 6e. The concentration of oxygen vacancies clearly increases with magnetic flux density.

The high-resolution Co 2p spectra contain two main peaks that correspond to Co 2p3/2 and 2p1/2 and two shake-up satellites at higher binding energy. This means that high-spin Co^2+^ ions were surrounded by O ions. In addition, for the film oxidized without an applied magnetic field, the energy difference between the two Co 2p peaks is 15.58 eV, which is similar to that of CoO (15.50 eV). This value is 15.29 eV for the film oxidized under an applied magnetic field of 6 T, which is lower than that of CoO and higher than that of Co metal (15.05 eV). This means that the amount of CoO in the film decreases when it is oxidized under a high magnetic field, which is consistent with the above finding that a high magnetic field inhibits oxidation of Zn. Additionally, the Co content of the film oxidized under an applied magnetic field of 6 T was higher than that of the film oxidized without an applied magnetic field, but Co substitution was not. Therefore, Co atoms may form clusters in the film oxidized under a high applied magnetic field. These results illustrate that a high magnetic field can be used to modify the state of Co in ZnO films.

**Figure 7:**
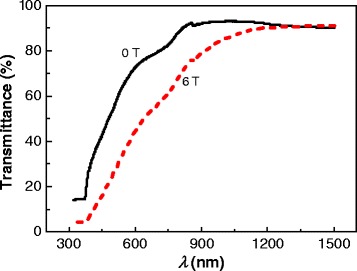
**Transmission spectra of Co-doped ZnO films oxidized with and without a high magnetic field.**

The optical transmittance of the Co-doped ZnO films is presented in Figure 7. The transmittance of the Co-doped ZnO film oxidized without an applied magnetic field is similar to reported results [25,26]. UV light (200 to 400 nm) was almost rejected completely by both films. The transmittance of the films increases with wavelength in the visible range (400 to 780 nm) and exceeds 80% for infrared light. However, for wavelengths lower than 1,200 nm, the transmittance of the film oxidized under an applied magnetic field of 6 T is lower than that of the film oxidized without an applied magnetic field. The transmittance is strongly related to the morphology of the films, the dendrite morphology aids transmittance.

The bandgap of films was calculated using the Tauc relation by extrapolating the linear portion of plots of (*αhυ*)^2^ against *hυ* to the (*αhυ*)^2^ = 0 axis [27], where *α* is the optical absorption coefficient and *hυ* is the incident photon energy. The calculated bandgaps of the films oxidized under magnetic fields of 0 and 6 T are 3.16 and 2.98 eV, respectively. These values are lower than that of pure ZnO (3.29 eV) prepared by thermal oxidation [28]. The bandgap of semiconductors is generally affected by the temperature and dopant. In this study, the bandgap only depends on the dopant because the bandgap was measured at RT in both cases. During thermal oxidation at 500°C, it is easy to dope the film with hydrogen to form an impurity band and reduce the effective bandgap [29]. Furthermore, the oxidation temperature was higher than that of pure ZnO (447°C). As a result, the bandgap of the films is smaller than that of pure ZnO because hydrogen may be readily introduced. Additionally, the bandgap of the film oxidized with an applied magnetic field of 6 T is smaller than that of the film oxidized without an applied magnetic field. This is because the greater density of oxygen vacancies in the film oxidized under a high magnetic field raises the impurity band, which narrows the bandgap.

**Figure 8:**
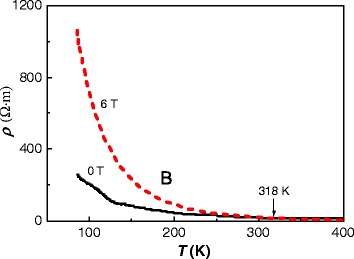
**Electrical resistivity** ***ρ*** **against temperature** ***T*** **of Co-doped ZnO films oxidized under magnetic fields of 0 and 6 T.**

The electrical resistivity of the films was investigated by recording their resistivity *ρ* at different temperatures *T*, as shown in Figure 8. The films behave as semiconductors because *ρ* decreases with increasing *T. ρ* of the film oxidized with an applied magnetic field of 6 T is higher than that of the film oxidized without an applied magnetic field when the temperature is lower than 318 K. According to a previous study, *ρ* is related to the concentration of oxygen vacancies and grain size [30]. Usually, a small grain size results in a high *ρ*, and a lower concentration of oxygen vacancies will result in a low *ρ*. However, in this study, a high concentration of vacancies leads to a high *ρ* because the scattering is enhanced by oxygen vacancies [31]. This result in the film formed under a magnetic field of 6 T having a higher *ρ* than that of the film oxidized without a magnetic field because the former contains more vacancies than the latter. The difference of *ρ* between the films is larger at lower *T*. High *ρ* can result from low carrier concentration and/or mobility [32]. In this study, because both films have no change of carrier concentration, the higher *ρ* of the films with deceasing *T* mainly results from lowered mobility. At lower temperature, the mobility of carriers is decreased. Because the concentration of oxygen vacancies is higher in the film formed under a magnetic field of 6 T than that formed under 0 T, more carriers are inactive at lower *T*. This leads to a large decrease in the mobility of carriers with decreasing *T*. Therefore, the difference in *ρ* between the two films increases with decreasing *T*. At RT, *ρ* of the film oxidized without a magnetic field is 0.164 Ω · m and that of the film oxidized under a magnetic field of 6 T is 0.813 Ω · m, both of which are much lower than that of an undoped ZnO film (1.429 Ω · m).

**Figure 9:**
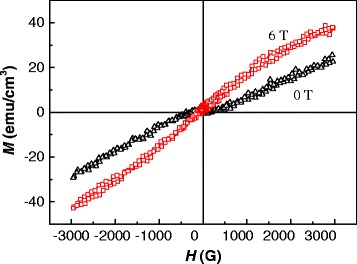
**Magnetization versus magnetic field (** ***M*** **-** ***H*** **) curves of Co-doped ZnO films oxidized with and without a high magnetic field.**

Plots of magnetization versus magnetic field (*M*-*H*) for the Co-doped ZnO films at RT are illustrated in Figure 9. Because the diamagnetic background was not subtracted, the films are difficult to magnetize to saturation. Both films exhibit well-defined magnetization hysteresis, implying that they are ferromagnetic at RT. The magnetism of the film oxidized under a magnetic field of 6 T is higher than that of the film oxidized without an applied magnetic field. This enhancement of magnetism is caused by the presence of oxygen vacancies and Co clusters in the film oxidized under a high magnetic field.

## Conclusions

This study investigating the effect of a high magnetic field on the oxidation of Co-doped ZnO films revealed that a high magnetic field is an effective tool to modify the structure, optical, electrical, and magnetic properties of Co-doped ZnO films without any contact. For the film oxidized without an applied magnetic field, ZnO was formed as nanowires by oxidation at the apexes of Zn particles, which was suppressed by the high magnetic field because it exerted a Lorentz force on the oxygen ions. This led to obvious differences in the surface morphologies of the films oxidized with and without an applied magnetic field, dendrite-like for the latter and polyhedral for the former. These variations of morphology markedly changed the optical transmittance of the films. Additionally, the Co substitution, concentration of oxygen vacancies and Co state in the Co-doped ZnO thin films were also affected by the application of a high magnetic field during oxidation. The increased concentration of oxygen vacancies caused by the application of a high magnetic field led to this film displaying different resistivity behavior compared with that of the film oxidized without a magnetic field. Furthermore, the variations of the concentration of oxygen vacancies and Co state under the high magnetic field increased the ferromagnetism of the film at RT. Therefore, a high magnetic field can be used to modify the structure and properties of ZnO thin films.

## References

[1] Dietl T, Ohno H, Matsukura F, Cibert J, Ferrand D (2000). Zener model description of ferromagnetism in zinc-blende magnetic semiconductors. Science..

[2] Özgür Ü, Alivov YI, Liu C, Teke A, Reshchikov MA, Doğan S (2005). A comprehensive review of ZnO materials and devices. J Appl Phys..

[3] Pan F, Song C, Liu XJ, Yang YC, Zeng F (2008). Ferromagnetism and possible application in spintronics of transition-metal-doped ZnO films. Mater Sci Eng R..

[4] Pearton SJ, Norton DP, Ip K, Heo Y, Steiner T (2005). Recent progress in processing and properties of ZnO. Prog Mater Sci..

[5] Klingshirn C, Fallert J, Zhou H, Sartor J, Thiele C, Mairer-Faig F (2010). 65 years of ZnO research - old and very recent results. Phys Status Solidi B..

[6] Li Q, Wang Y, Fan L, Liu J, Kong W, Ye B (2013). Coexistence of superparamagnetism and ferromagnetism in Co-doped ZnO nanocrystalline films. Scripta Mater..

[7] Ramasubramanian S, Thangavel R, Rajagopalan M, Thamizhavel A, Asokan K, Kanjilal D (2013). Study on the ferromagnetism in Co and N doped ZnO thin films. Curr Appl Phys..

[8] Subramanian M, Tanemura M, Hihara T, Ganesan V, Soga T, Jimbo T (2010). Magnetic anisotropy in nanocrystalline Co-doped ZnO thin films. Chem Phys Lett..

[9] Kaushik A, Dalela B, Rathore R, Vats VS, Choudhary BL, Alvi PA (2013). Influence of Co doping on the structural, optical and magnetic properties of ZnO nanocrystals. J Alloy Compd..

[10] Vagadia M, Ravalia A, Katba S, Solanki PS, Bapna K, Kumar M (2014). Co-substitution driven electronic structure modifications in Zn_1-x_Co_x_O. J Alloy Compd..

[11] Li DY, Zeng YJ, Pereira LMC, Batuk D, Hadermann J, Zhang YZ (2013). Anisotropic magnetism and spin-dependent transport in Co nanoparticle embedded ZnO thin films. J Appl Phys..

[12] Shi S, Yang Y, Xu J, Li L, Zhang X, Hu G (2013). Structural, optical and magnetic properties of Co-doped ZnO nanorods prepared by hydrothermal method. J Alloy Compd..

[13] Bilecka I, Luo L, Djerdj I, Rossell MD, Jagodič M, Jagličić Z (2011). Microwave-assisted nonaqueous sol–gel chemistry for highly concentrated ZnO-based magnetic semiconductor nanocrystals. J Phys Chem C..

[14] Rambu AP (2012). The influence of oxidation time on the properties of oxidized zinc films. Superlattice Microst..

[15] Gu H, Zhang W, Xu Y, Yan M (2012). Effect of oxygen deficiency on room temperature ferromagnetism in Co doped ZnO. Appl Phys Lett..

[16] Alaria J, Venkatesan M, Coey JMD (2008). Magnetism of ZnO nanoparticles doped with 3d cations prepared by a solvothermal method. J Appl Phys.

[17] Li GJ, Cao YZ, Wang Q, Du JJ, Zhao Y, He JC (2014). Tunable phase formation in Ni-Fe thin films at nanoscale using high magnetic field. Vacuum..

[18] Li GJ, Du JJ, Wang HM, Wang Q, Ma YH, He JC (2014). High magnetic field induced pillar growth and subsequent magnetic properties of the thermal evaporated Co thin films. Mater Lett..

[19] Wang Q, Cao YZ, Li GJ, Wang K, Du JJ, He JC (2013). Improving the magnetic properties of molecular-beam-vapor-deposited Ni_45_Fe_55_ nanocrystalline films by in-situ high magnetic field applications. Sci Adv Mater..

[20] Cao YZ, Wang Q, Li GJ, Du JJ, Wu C, He JC (2013). Effects of high magnetic field on the structure evolution, magnetic and electrical properties of the molecular beam vapor deposited Fe_*x*_Ni_1-*x*_ (0.3 ≤ *x* < 0.8) thin films. J Magn Magn Mater.

[21] Du JJ, Li GJ, Wang Q, Cao YZ, Ma YH, He JC (2014). Effects of high magnetic field on the structural evolution and magnetic properties of nanocrystalline Ni films. Nano.

[22] Kim TW, Kawazoe T, Yamazaki S, Ohtsu M, Sekiguchi T (2004). Low-temperature orientation-selective growth and ultraviolet emission of single-crystal ZnO nanowires. Appl Phys Lett..

[23] Kasuga M, Takano T, Akiyama S, Hiroshima K, Yano K, Kishio K (2005). Growth of ZnO films by MOCVD in high magnetic field. J Cryst Growth..

[24] Wagner CD, Riggs WM, Davis LE, Moulder JF, Muilenberg GE (1979). Handbook of X-ray photoelectron spectroscopy.

[25] Benramache S, Temam HB, Arif A, Guettaf A, Belahssen O (2014). Correlation between the structural and optical properties of Co doped ZnO thin films prepared at different film thickness. Optik..

[26] Rambu AP, Rusu GI (2010). Effect of preparation conditions on the microstructural characteristics and optical properties of oxidized zinc films. Superlattice Microst..

[27] Ivill M, Pearson SJ, Rawal S, Leu L, Sakik P, Das R (2008). Structure and magnetism of cobalt-doped ZnO thin films. New J Phys..

[28] Rusu GG, Râmbu AP, Buta VE, Dobromir M, Luca D, Rusu M (2010). Structural and optical characterization of Al-doped ZnO films prepared by thermal oxidation of evaporated Zn/Al multilayered films. Mater Chem Phys..

[29] Huang XH, Zhan ZY, Pramoda KP, Zhang C, Zheng LX, Chua SJ (2012). Correlating the enhancement of UV luminescence from solution-grown ZnO nanorods with hydrogen doping. CrystEngComm..

[30] Soumahoro I, Colis S, Schmerber G, Leuvrey C, Barre S, Ulhaq-Bouillet C (2014). Structural, optical, spectroscopic and electrical properties of Mo-doped ZnO thin films grown by radio frequency magnetron sputtering. Thin Solid Films..

[31] Li B, Zhu H, Liu Q, Liu Z, Zhang Y (2014). Low temperature electrical transport behavior of La_0.7_Ba_0.3_MnO_3_ thin films on LaAlO_3_ substrate. J Magn Magn Mater..

[32] Das AK, Misra P, Kumar R, Ganguli T, Singh MK, Phase DM (2014). Studies on highly resistive ZnO thin films grown by DC-discharge-assisted pulsed laser deposition. Appl Phys A..

